# Enhancing accuracy in proton therapy: The impact of geometric uncertainty models in head and neck cancer treatment

**DOI:** 10.1002/mp.17698

**Published:** 2025-02-21

**Authors:** Ying Zhang, Mark Ka Heng Chan

**Affiliations:** ^1^ Department of Medical Physics and Biomedical Engineering University College London London UK; ^2^ Department of Radiation Oncology University Nebraska Medical Center Omaha USA

**Keywords:** application of anatomical modeling, head and neck cancer, intensity‐modulated proton therapy, robust optimization, uncertainty management

## Abstract

**Background:**

Anatomical changes present a major source of uncertainty in head and neck (H&N) cancer treatment. Accurate modeling of these changes is important for enhancing treatment precision and supporting better outcomes.

**Purpose:**

The purpose of this study is to assess different anatomical uncertainty modeling methods in robust optimization for H&N cancer proton therapy.

**Methods:**

This retrospective study involved five nasopharynx radiotherapy patients. We compared conventional robust optimization with anatomical robust optimization (aRO): (1) conventional robust optimization (cRO‐3 mm), which used 3 mm setup shift and 3% range uncertainty. (2) aRO_AM which used three predicted images from an AM capturing systematic anatomical changes, with a 1 mm setup shift and 3% range uncertainty. (3) aRO_PM, which used three predicted images from a probability model (PM) capturing the most probable deformations, also with a 1 mm setup shift and 3% range uncertainty. We assessed weekly dose coverage of the clinical target volumes (CTVs). Normal tissue complication probability (NTCP) for grade ≥ 2 xerostomia and grade ≥ 2 dysphagia were calculated using the accumulated nominal dose (without errors).

**Results:**

aRO_PM outperformed cRO‐3 mm and aRO_AM, consistently achieving V94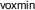 ≥ 95% for all cases across treatment weeks. Additionally, aRO_PM reduced the NTCP for grade ≥2 xerostomia by an average of 4.88 %, with a maximum reduction of 8.03%, and reduced the NTCP for grade ≥2 dysphagia by an average of 1.80%, with a maximum reduction of 4.23 %.

**Conclusion:**

The PM demonstrates potential for improving robust optimization by effectively managing anatomical uncertainties in H&N cancer proton therapy, thereby enhancing treatment effectiveness.

## 1 INTRODUCTION

Proton therapy has shown the potential in minimizing radiation dose to the normal tissue,^1^ compared to conventional photon therapy. However, this precise delivery technique has inherent sensitivity to various uncertainties. In head and neck (H&N) treatment, geometric uncertainties are common throughout the treatment course and include factors such as beam reproducibility, patient positioning error and anatomical changes. Anatomical changes can include variations in nasal filling, jaw movement, neck folds, spine flexion, shoulder positioning, as well as tumor shrinkage, gland shrinkage, and patient weight loss.^2^, ^3^

In conventional robust planning for H&N cancer, geometric uncertainty is modeled by rigid shifts of 3 mm. However, anatomical changes that occur during treatment are often not uniform in all directions. Thus, applying the same 3 mm shift may inadvertently result in excessive radiation dose to the organs at risk (OARs).

Image‐guided radiotherapy (IGRT) has improved position management by allowing six‐degree‐of‐freedom corrections in real time, and some institutions have shown that the residual position uncertainty can be reduced to about 1 mm.^4^, ^5^

Previously, average‐model (AM)‐based robust optimization has been used to include systematic anatomical changes in treatment planning. Yet, non‐rigid positioning variations can still cause significant dosimetric discrepancies.^2^ In this study, we exploited the probability model (PM)^6^ to account for both systematic progressions and random deformations. By identifying the most probable anatomical changes, we incorporated them into robust optimization. We then compared anatomically robust plans optimized using the PM, against both AM and conventional robust plans, focusing on potential improvements in tumor coverage and normal tissue sparing.

## 2 MATERIALS AND METHODS

### 2.1 Patient data

We obtained weekly deformation data from a total of 20 advanced nasopharyngeal carcinoma patients. Details regarding the patients' diseases, original treatments, and evaluation of anatomical changes can be found in refs. [7, 8, 9, 10]. Contoured CT imaging data of five patients randomly selected were included in this study. It should be noted that this is a retrospective study using the patients' imaging data and contours. Each patient had a planning CT (pCT), which is also referred to as week 0, and weekly CT (wCT) from week 1 to 6 (at fractions of 5, 10, 15,…, 30).

### 2.2 Anatomical models

Using a leave‐one‐out cross‐validation approach, we built two models based on 20 patients obtained. For the AM, we predicted deformation for each patient by applying the average deformation from the training group (*n* = 19) to the patient's pCT. For the PM, we estimated the probability distribution of random deformations and applied the most probable random deformation, combined with the average deformation, to the patient's pCT. The details of the AM and PM and model evaluation can be found in refs. [11] and [6]. This process was iterated for five randomly selected patients. The AM and PM, which were built at each time point, was used to generate predicted images of weeks 1, 3 and 5 for these five patients.

### 2.3 Treatment planning

Three proton plans with different optimization scenarios are implemented in this study. (1) cRO‐3 mm: we applied 14 setup error scenarios of 3 mm shifts (6 principal directions and 8 from the center to the vertices, illustrated in Appendix A) and 2 range error scenarios of ±3 % to the pCT, resulting in 28 total uncertainty scenarios. (2) aRO_AM: we extended these same 28 uncertainty scenarios to the pCT and three predicted CTs at weeks 1, 3, and 5 of the AM. For aRO_AM, a 1 mm setup shift and a 3% range error were used. (3) aRO_PM: Similarly, we extended the same 28 scenarios to the pCT and three predicted CTs at weeks 1, 3, and 5 of the PM, again with a 1 mm setup shift and a 3% range error. The total number of uncertainty scenarios in aRO (whether AM or PM) increases to 4 × 14 × 2  = 112.

The prescription dose for the low‐risk clinical target volume (CTV) and the high‐risk CTV are 54.25 and 70 Gy, respectively. Plans were optimised under the corresponding uncertainty scenarios with the following constraints to both CTVs: $V94_{\text{voxmin}}$ ≥ 98% and D2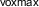 ≤ 110% of the prescribed dose, where voxmin dose distribution records the minimum dose of each voxel under uncertainty scenarios, and voxmax dose distribution records the maximum dose of each voxel under uncertainty scenarios. Replanning will be triggered when CTV $V94_{\text{voxmin}}<$ 95%. For serial OARs, we defined constraints based on the voxmax: D0.03${\mathrm{cc}}_{\text{voxmax}}$ (The voxmax dose to the hottest 0.03 ${\mathrm{cm}}^{3}$) <68 Gy for brainstem, D0.03${\mathrm{cc}}_{\text{voxmax}}$ <58.5 Gy for spinal cord, and D0.03${\mathrm{cc}}_{\text{voxmax}}$ <64 Gy for optic nerves and chiasm. For other parallel OARs, our goal is to minimize the dose as much as possible through maximizing the delta normal tissue complication probabilities (NTCPs) of grade ≥2 patient‐rated xerostomia and dysphagia by comparison with the reference photon plan according to the Dutch model‐based selection of proton and photon radiotherapy for H&N cancer patients. The planning details and detailed dosimetric table can be found in Appendix A.

#### 2.3.1 Plan evaluation using accumulated dose metrics

We simulated the effects of 3% range uncertainty and 1 mm residual position uncertainty for each wCT. This process generates 28 dose distributions for each wCT. Then, we calculated the voxmin and voxmax from the total 28 scenarios. Anaconda deformable image registration of RayStation was used to accumulate the voxmin, voxmax, and nominal dose in the planning frame as the accumulated voxmin, accumulated voxmax, and accumulated nominal dose.

The accumulated mean dose (Dmean) of bilateral submandibular and parotid glands was converted to normal tissue complication probabilities (NTCP) for grade ≥ 2 patient‐rated xerostomia (NTCP_x) and Dmean of oral cavity and pharyngeal constrictor muscle (PCM) superior, medius and inferior was used for NTCP of grade ≥2 physician‐rated dysphagia (NTCP_d) according to the validated models of the national indication protocol for proton therapy in the Netherlands for H&N cancer,^12^ Appendix B includes the equation details.

## 3 RESULTS

**FIGURE 1:**
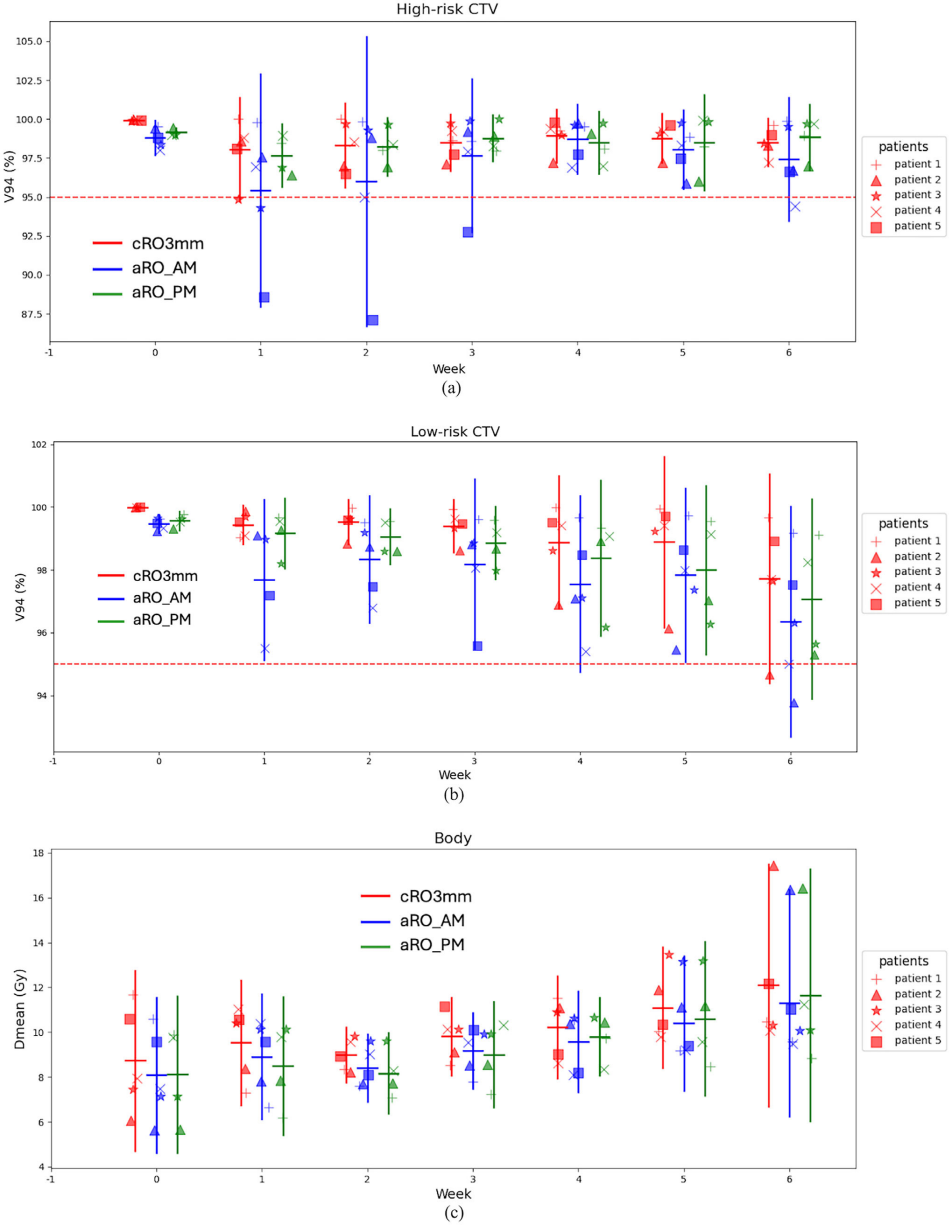
The weekly comparison of three different strategies on CTV coverage and body mean dose on five patients. The dashed lines indicate the replanning threshold.The horizontal line in the scatter data represents the mean value of the distribution, while the vertical line spans over its 95% confidential interval. CTV, clinical target volume.

Figure 1 presents the individual weekly $V94_{\text{voxmin}}$ of CTVs and mean dose of body across three different strategies. For CTV coverage, in all scenarios for the five patients, aRO_PM maintained good CTV coverage above the replan‐triggering threshold. In contrast, aRO_AM failed in eight scenarios (from patients 2, 3, 4, and 5), while cRO‐3 mm failed in two scenarios (from patients 2 and 3). When examining the mean $V94_{\text{voxmin}}$ values, cRO‐3 mm starts with a higher planning value than aRO_PM. However, for the high‐risk CTV, the mean value of aRO_PM gradually surpasses cRO‐3 mm starting from week 2. For the low‐risk CTV, the mean value of aRO_PM remains slightly below cRO‐3 mm overall, but considering the initial gap, the largest difference is only ‐0.47%. Overall, aRO_PM provided more consistent tumor coverage. For body mean dose, cRO‐3 mm has the highest body Dmean, followed by aRO_PM and aRO_AM.

**FIGURE 2:**
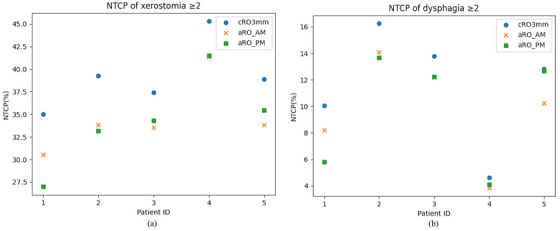
The comparison between the NTCPs of cRO‐3 mm, aRO_AM and aRO_PM on for the five validation patients. (a) The NTCP of grade ≥2 patient‐rated xerostomia. (b) The NTCP of grade ≥2 patient‐rated dysphagia. NTCP, normal tissue complication probability.

We evaluated the NTCP_x and NTCP_d for each patient in Figure 2. Compared with cRO‐3 mm, the maximum reduction in the NTCP_x by using the aRO_PM strategy is 8.03% with an average reduction of 4.88%. The maximum reduction in the NTCP_d by using the aRO_PM strategy is 4.23%, with an average reduction of 1.80%.

The original data of the planning, weekly dose and accumulated dose for each patient are listed in the supplementary data.

## 4 DISCUSSION

This study evaluates three proton therapy optimization strategies, cRO‐3 mm, aRO_AM, and aRO_PM to address geometric uncertainty in H&N cancer treatment. The aRO_PM approach showed more consistent tumor coverage and reduced NTCP compared to the conventional cRO‐3 mm method, especially under variable anatomical conditions.

In this study, the aRO_PM demonstrated superior tumor coverage compared to aRO_AM. However, this increased robustness comes at the cost of slightly reduced NTCP benefits. The difference in mean body dose between aRO_PM and aRO_AM was minimal on average.

Chan et al.^13^ compared anatomical robust optimization using predicted images of AM from 1, 3, and 5 against 2 and 4. Their results indicate that using more predicted images can lead to more robust treatment plans. In future work, we will investigate different strategies to identify the most appropriate time points for presenting typical anatomical changes. Notably, the anatomical model is not limited to nasopharyngeal cancer; it can also be applied to other H&N cancers such as oral, oropharyngeal, or laryngeal cancer.

Increasing the number of uncertainty scenarios from 28 (cRO) to 112 (aRO) inevitably prolongs the optimization process, as each additional scenario must be computed and integrated into the final plan. In our experience, an aRO plan, which includes the pCT plus three predicted CTs, takes about 18 min for 30 iterations, compared with two minutes for a single CT in cRO. Since 5–6 optimization rounds are often needed for aRO (vs. 2–3 for cRO), the total aRO planning time can reach 90 min‐roughly 18 times longer than single‐CT planning. Despite this higher computational cost, the improved robustness from simultaneously addressing multiple anatomical variations may justify the additional effort in high‐precision H&N proton therapy.

Online adaptation has demonstrated potential for reducing NTCP in proton therapy. Lalonde et al.^14^ reported mean NTCP reductions of 5.42% (NTCP_d) and 5.12% (NTCP_x) compared with the cRO‐3 mm approach, based on an assumption of perfect setup without error during IGRT. In our study, we used a 1 mm setup margin to account for robotic table correction accuracy and beam reproducibility, achieving a comparable NTCP_x reduction of 4.88% relative to cRO‐3 mm. NTCP_d reduction was limited, however, due to significant overlap between the superior portion of the PCM and the high‐risk CTV, making it difficult to spare small non‐overlapping areas in these cases. Moreover, online adaptation requires a median of 12 min (ranging from 8 to 22 min) per session. During this adaptation period, patients may experience posture changes, which our study found could lead to 10% variations in CTV V94 and changes in OAR mean dose of up to 8.5 Gy, as evidenced by comparing week 1 treatment doses with planning doses (please refer to appendix table). Thus, the benefits reported by Lalonde et al. may not guarantee. By contrast, the time saved per patient using the aRO_PM approach may offer an advantage in improving overall treatment efficiency. It would be interesting to compare the aRO_PM with online adaptation in the future work.

## CONFLICT OF INTEREST STATEMENT

The authors have no relevant conflicts of interest to disclose.

## Supporting information

Supporting Information

Supporting Information

## Data Availability

The authors cannot make these data publicly available due to data use agreement.
