# Supplementary material for: Enhancing accuracy in proton therapy: The impact of geometric uncertainty models in head and neck cancer treatment
Source: Med Phys. 2025 Feb 21;52(6):4585–9. doi: 10.1002/mp.17698 (PMC12149682; doi:10.1002/mp.17698)
Supplement: Supplementary file 1 — Supporting Information [file MP-52-4585-s002.pdf]

Appendix A: Robust optimisation details

Figure 1) illustrates the 14 setup error scenarios used in robust optimisation.

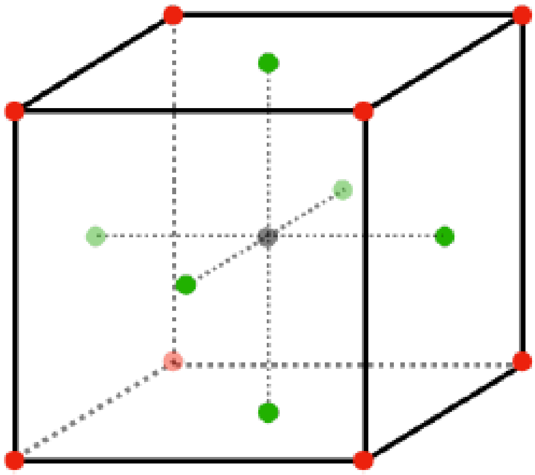

FIG. 1: The setup error in directions. 6 principal directions (6 green dots) and from the centre to the vertices(8 red dots), the combinations of these including ±3% range uncertainty result in 28 scenarios.

In the conventional robust optimisation, we applied 14 setup error scenarios of 3 mm shifts (see Figure 1) and 2 range error scenario of ±3 % to the planning CT, giving a total of 28 uncertainty scenarios.

In anatomical robust optimisation, the same 28 uncertainty scenarios are applied to four image sets - the planning CT plus three predicted CTs. the total number of uncertainty scenarios increases to $4 \times 14 \times 2$ =112.

The model is most effective in predicting the patient outline changes. Hence, in robust optimisation, the contours on the predicted images for robust optimisation are created by following theses rules: For all OAR contours and the low-risk CTV (nodal area) affected by neck changes, we use the predicted contours from contour propagation. For the high-risk CTV, we use the initial CTV of the planning CT in the predicted plan to ensure target coverage. To be noted, the contours on the predicted CT would be used in the robust function.

A standard six-beam arrangement for NPC was adopted [1], comprising two anterior obliques beams(gantry 45 and 315), two lateral beams(gantry at 90 and 270 with 10-degree deviation and possible couch rotation of 10 to 15 degrees) beams with a 4cm range shifter, and two posterior obliques beams(gantry at 160 and 200). In addition, various beam-specific blocks, including sinus and amalgam dental filling blocks for anterior beams, and shoulder blocks for posterior beams, were used to mitigate the range uncertainty due to anatomical variations. More details are described in [1].

The dosimetric goals for all plans in this study are summarised in Table I for CTVs and serial organs [2].

TABLE I: Dosimetric goals of the treatment plans created in this study

Structure	Clinical goal under 3mm/3% uncertainty	
high-risk CTV (Prescription: 70Gy, 35 fractions)	$V94_{voxmin}$(The percentage of CTV volume received at least 94% of prescription dose in voxmin dose distribution) > 98%	1
low-risk-CTV (Prescription: 54.25Gy, 35 fractions)	$V94_{voxmin}$ > 98%	1
CTV	$D2_{voxmax}$ (The minimum dose to the hottest 2% volume) < 110% of prescription dose (77Gy)	1
Brainstem	$D0.03cc_{voxmax}$ (The voxmax dose to the hottest 0.03 cubic centimetre) <68 Gy	2
Spinal cord	$D0.03cc_{voxmax}$ <58.5 Gy	2
Optic nerves and chiasm	$D0.03cc_{voxmax}$ <64 Gy	2
**Structure**	**Clinical goal without uncertainty**	**Priority**
Brainstem	D0.03cc (The nominal dose to the hottest 0.03 cubic centimetre)<63.1 Gy	2
Spinal cord	D0.03cc <55 Gy	2
Optic nerves and chiasm	D0.03cc <59.5 Gy	2

All plans were generated in a research version of RayStation (v.11B, RaySearch, Stockholm, Sweden) for the IBA Proteus Plus machine, leveraging pencil beam spot scanning technology. The minimax robust optimization algorithm will search for a solution where the resulting robust objectives can be achieved as far as possible in all these scenarios (different anatomical CTs, setup error and range error), such as brainstem dose constraints need to be satisfied on the predicted CT using its predicted contours. The details of robust optimisation algorithm can be found in the official document of Raystation.

Appendix B: Normal tissue complication probability models

$$NTCP = (1 + \exp(-S))^{-1} \tag{B1}$$

$S_{xerostomia}$ for xerostomia and sticky saliva is given by S(patient-rated xerostomia) = -2.2951+0.0182*(mean dose submandibular) + 0.0996*($\sqrt{}$ mean dose ipsilateral parotid + $\sqrt{}$ mean dose contralateral parotid).

$S_{dysphagia}$ for dysphagia is given by S(patient-rated dysphagia) = -4.0536+0.03*(mean dose oral cavity) + 0.0236*(mean dose PCM superior) + 0.0095 * (mean dose PCM medius) + 0.0133 * (mean dose PCM inferior) - 0.6281 * (if tumour location is pharynx).

---

[1] Daniel Scandurra, Tineke W.H. Meijer, Jeffrey Free, Johanna G.M. van den Hoek, Lotta Kelder, Edwin Oldehinkel, Roel J.H.M. Steenbakkers, Stefan Both, and Johannes A. Langendijk. Evaluation of robustly optimised intensity modulated proton therapy for nasopharyngeal carcinoma. *Radiotherapy and Oncology*, 168, 2022.

[2] Erik W. Korevaar, Steven J.M. Habraken, Daniel Scandurra, Roel G.J. Kierkels, Mirko Unipan, Martijn G.C. Eenink, Roel J.H.M. Steenbakkers, Stephanie G. Peeters, Jaap D. Zindler, Mischa Hoogeman, and Johannes A. Langendijk. Practical robustness evaluation in radiotherapy – A photon and proton-proof alternative to PTV-based plan evaluation. *Radiotherapy and Oncology*, 141, 2019.
